# The evolution of Gaia(s)

**DOI:** 10.1098/rstb.2024.0095

**Published:** 2025-08-07

**Authors:** Timothy M. Lenton

**Affiliations:** ^1^ Global Systems Institute, University of Exeter, Exeter EX4 4QE, UK

**Keywords:** Gaia, biosphere, entropy, evolution, selection, systems

## Abstract

The Earth’s biosphere, or ‘Gaia’, has increased its own persistence and flourishing through intense global cycling of essential elements, and net stabilization of atmospheric composition and climate. A key question is how has it acquired these remarkable properties? Did it involve some form of natural selection among parts of the biosphere? (And if so; which parts?) Or could these properties arise another way? A physical explanation is that in a complex system containing a source of variation and some ‘memory’ of past states, the most probable trajectory is towards a state of greater size, diversity and stability. Models show this state tends to be reached through a series of progressively more stable configurations, separated by system reorganizations, which become more difficult and thus less frequent over time. Such Gaia-level evolution can also be interpreted in terms of selection among ecosystems and nutrient cycles based on how well they persist and/or spread. Thus, what seem very different physical and biological paths to explaining Gaia are converging. The resulting theory makes testable predictions about how Earth’s biosphere evolved that are consistent with available evidence. It also makes predictions of how biospheres in general evolve, which will become testable if/when exo-biospheres are detected.

This article is part of the discussion meeting issue ‘Chance and purpose in the evolution of biospheres’.

## 1. Introduction

Life (the clade of all extant biota) on Earth has increased its own persistence and flourishing in several crucial ways [1]: over time Earth’s biosphere has tended towards greater free energy capture (productivity) [2,3], more intense cycling of essential elements [4], increasing diversity [5], declining extinction rates [5,6], the origin of stabilizing mechanisms for variables such as atmospheric oxygen and ocean nutrients [7,8] and the augmentation of stabilizing mechanisms for other variables such as atmospheric carbon dioxide and global temperature [9,10]. The journey has not been smooth—Life has sometimes destabilized its planetary environment (as humas are doing now), and occasionally this has led to system-wide reorganizations, producing an overall pattern of ‘punctuated equilibria’ [11,12]. Nevertheless, in a probabilistic average sense there has been a positive net trend towards improved habitability, which I term the Gaia phenomenon. The task is to try to explain it. Beyond appealing to the weak anthropic principle—that we would not be here to observe these properties unless they were in place [13]—an evolutionary explanation is needed, meant in the broad sense of a mechanistic explanation of how these properties could have developed over time [12].

The original Gaia hypothesis [14–17] of ‘atmospheric homeostasis by and for the biosphere’ first recognized global stabilizing properties and attributed them to Life, but did not explain how they had come about. Or rather, by describing atmospheric homeostasis teleologically as a purposive product of Life it was taken to be akin to an adaptation of an organism and therefore the product of natural selection. When evolutionary biologists realized this, they pointed out that natural selection could not be working on a population of one biosphere, and they argued it could not act at lower levels to produce stabilizing properties at the biosphere scale [18,19]. They framed Gaia as if it were a giant result of altruism—organisms paying a fitness penalty to create a better environment for all—that would be vulnerable to ‘cheats’ that would disrupt regulation [18–20].

This was never the hypothesis [21], and in response, a consensus emerged that Gaia must principally be built on the by-products of metabolisms originally selected for other reasons [4,22,23]. These by-products include, for example, the production of O_2_ (a waste product of oxygenic photosynthesis) and of CO_2_ (the result of aerobic respiration). Models showed that long-term regulation of well-mixed atmospheric gases such as these can be explained by (negative) feedback on the collective growth of the diverse community affecting them, without involving natural selection [22]. In other cases, by-products could have differential environmental effects that feed back to affect natural selection [22]. An example is the release of dimethyl-sulfide (DMS) by marine algae resulting in localized increases in cloud condensation nuclei and cloud reflectivity [22]. Such effects could conceivably become adaptive at higher levels of selection [24], in this example through clonal patches of algae enhancing the atmospheric dispersal of a few of their members [25]. Meanwhile, while the focus of the debate had been on the origin of stabilizing properties (homeostasis), it was recognized that Gaia’s extraordinary internal recycling properties deserved greater explanatory effort [4]. Subsequent models showed how natural selection can robustly produce nutrient recycling [26]—even it is altruistic [27]—and that a form of ecosystem-level selection could produce regulation of heterogeneous environmental variables like temperature [28,29].

Recently there has been a (modest) resurgence of interest in trying to explain the evolution of Gaia, especially trying to reconcile it with a generalized form of natural selection [30–36], while a different approach invokes principles from cybernetics, statistical physics and non-equilibrium thermodynamics [30,37–39]. Here I synthesize this research into different physical and biological paths to explaining Gaia, and I argue that they are (finally) starting to converge. I start by tackling some widespread misnomers about Gaia, because this opens the space of explanations that can be plausibly entertained. Then I follow each path in turn, staring with the less well trod physical path.

## 2. Gaia need not be an organism with adaptations

The originator of the Gaia hypothesis James Lovelock sometimes metaphorically likened Gaia to an organism [11,40], but as his collaborator Lynn Margulis put it ‘no organism eats its own waste products’ [41]. Organisms continually exchange materials (as well as energy) with their surroundings as a key part of staying alive. Gaia by contrast is nearly materially closed. There is only a tiny input of matter from space and loss of hydrogen to space, and the exchange of materials with the inner Earth, via volcanism and plate tectonics, is dwarfed by the cycling of materials within the system [1,4]. This is clearly seen in the massive exchange fluxes of gases between the Earth’s surface and the atmosphere [22]. Organisms also exist in interacting populations, whereas if there is a population of biospheres they are not interacting.

Despite their differing views on the organism metaphor, Lovelock and Margulis started asking purposive questions of aspects of the biosphere: ‘If we assume the Gaia hypothesis, and regard the atmosphere as a contrivance, then it is reasonable to ask what is the function of its various component gases. Outside the Gaia hypothesis, such a question would rightly be condemned as circular and illogical but in its context such questions are no more unreasonable than asking, for example, what is the function of fibrinogen in blood’ [15]. Here they explicitly recognize that such teleological language framed properties of Gaia as if they were adaptations. However, since the 19th century it has only been considered philosophically sound to attribute functions to artefacts (engineered for a purpose) or to organisms (evolved by a process of natural selection, which is taken to somehow replace the action of the engineer) [21]. Thus, the organism metaphor remained.

Natural selection is widely argued to be the ‘sole mechanism’ that can produce complex structures like the eye from random variation [42]. This has led some authors to suggest that natural selection is the only process that could produce the properties of Gaia in need of explanation [34,43]. But what are those key properties? Two stand out: the stabilization of aspects of atmospheric composition, climate and ocean composition that support persistence, and the intense global cycling of essential elements that supports greater productivity (flourishing). The extraordinary recycling within Gaia is quite distinct from anything exhibited by an organism, while the stabilizing properties are at best less precise than the homeostasis of an organism.

It is therefore not obvious that the explanation of these properties must necessarily follow the same framework one would use to rationalize adaptations of an organism. It may do, and that explanatory framework is certainly powerful. But here I first ask: How much can we explain without invoking (natural) selection and adaptation? In doing so, instead of likening Gaia to an organism, I follow Bruno Latour [44] in treating Gaia as a *sui generis* phenomenon (of its own kind).

## 3. The physical path to explaining Gaia

Cybernetics has long shown that when innovation within a dynamical system produces unstable system-level behaviour this tends to be short-lived, as the system will keep changing until it finds a stable configuration, which (by definition) tends to persist. The system may be physical or biological. In the language of dynamical systems: the system moves through its phase space (or configuration space), which describes all possible states of the system, sometimes encountering ‘repellers’ which accelerate change, until it settles in an ‘attractor’ (a set of states towards which a system tends to evolve). This is how a dynamical system can ‘find’ stability [45,46]. The search may be entirely random, but the landscape has structure which affects the temporal behaviour. Once in an attractor, if there is innovation within the system or external perturbation, it may get pushed out of the attractor. In the extreme case, where the resulting change destroys any ‘memory’ of past states or configurations, the system will be totally reset. Yet stability can still robustly be (re)found—albeit a different stability in each case.

Ross Ashby first described and demonstrated this mechanism in the late 1940s with a machine he called the ‘homeostat’ [45,46]. The mechanism does not require biology, although the machine clearly needed a living inventor, and Ashby was trying to explain the origin of self-regulatory behaviour in biology. The mechanism can explain how global stability can be found through a series of trials over time and it tends to produce a pattern of ‘punctuated equilibria’ as observed, e.g. in macroevolution [47] or the history of atmospheric oxygen [12]. It has been termed ‘sequential selection’ [30,48] to refer to selection of a system state through a series of trials over time (whereas natural selection can be thought of as selection from trials over both space and time).

In the extreme case of total resets of a system that destroy any ‘memory’ of past behaviour, there can be no overall tendency towards greater stability over time [38]—no ‘learning’. However, in real complex systems, including Gaia, that undergo change through punctuated equilibria, there is usually continuity of aspects of the system through time, i.e. some ‘memory’. In the presence of such ‘memory’, it may come as a surprise to learn that sequential selection can produce a general tendency for complex systems to increase in stability over time. This is a probabilistic feature, not a guarantee—on average, things get more stable. Nevertheless, it is fundamental. How can it happen? And what are the minimal features required? It does not require biology. Physical examples include spin glasses and magnetic relaxation in type II superconductors [49]. This is philosophically important because it offers a common explanatory framework for increasing stability across the non-living and the living realms—avoiding vitalism. It may thus help explain the origin of life. But here I retain the focus on Gaia.

**Figure 1.**
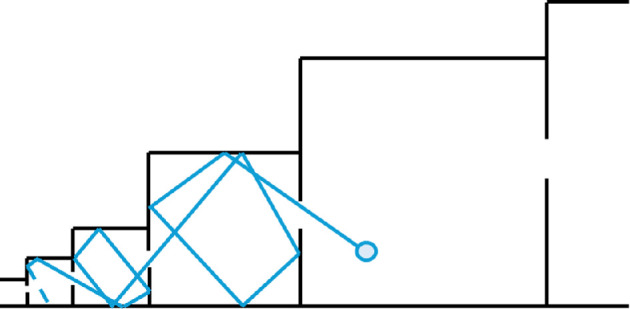
The entropic ratchet for the simple physical case of a particle (e.g. gas molecule) moving between progressively larger connected boxes—following Arthur & Nicholson [38]. The particle is most likely to be found in the macro-state (box) with the largest number of micro-states.

The most general explanation, from statistical mechanics, is that sufficiently complex systems evolve in the direction of increasing information entropy [38,50]. This simply means that the most probable overall (macro) state in which to observe a system is the one with the largest number of corresponding states of its components (micro-states). (Formally, the entropy of a macro-state is the log of the number of micro-states that can realize it.) This can be neatly illustrated [38] by the simple physical case of a particle (e.g. gas molecule) found in a series of progressively larger connected boxes (figure 1). Even if the particle starts in the smallest box, it is most likely to be found in the largest box (macro-state) where it has the most possible locations (micro-states). Here the movement from one micro-state to another is a random walk (through the configuration space of all possible micro-states), but the system tends towards macro-states characterized by larger numbers of micro-states (bigger boxes). The general phenomenon is termed an ‘entropic ratchet’ (or entropic hierarchy). The ‘entropic barrier’ to the particle leaving a larger box (through the window) is higher than for a smaller box, meaning that the system will tend to spend progressively longer in macro-states with a larger number of micro-states.

**Figure 2.**
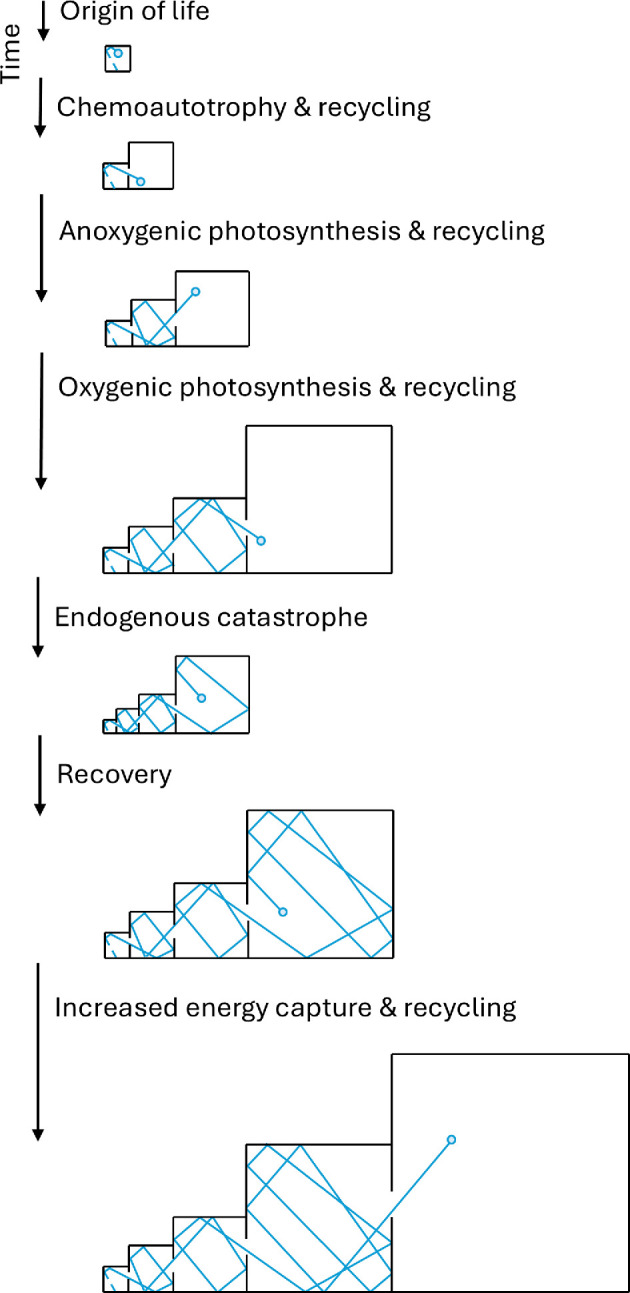
Schematic extension of the entropic ratchet (from figure 1) to a biosphere that can endogenously generate larger ‘boxes’ through the evolution of new metabolisms of energy capture and resource recycling, and endogenously shrink or expand the ‘boxes’ through e.g. effects on the abiotic environment.

For Gaia, a micro-state is a particular combination of diverse living and non-living things, while the macro-state includes aggregate properties like total productivity, mean temperature, atmospheric composition, etc. [38]. While each possible combination of organisms gives rise to a particular global macro-state, each state of the global environment can be realized by many different combinations of organisms. Crucially, however, some macro-states have a larger number of corresponding micro-states than others. A small and simple biosphere has relatively few micro-states, a large and complex biosphere has many micro-states. An important addition to the boxes metaphor for a biosphere is that it can endogenously generate larger ‘boxes’ (macro-states with a larger number of micro-states). It can do this, for example, through the evolution of more effective metabolisms of free energy capture (e.g. forms of photosynthesis) and more efficient pathways of resource recycling (figure 2). Of course, biology can also endogenously shrink the ‘boxes’, e.g. by driving the environment towards a less habitable state (e.g. ‘snowball Earth’). However, it will tend to spend less time in such macro-states.

In general, a complex system may tend to get ‘stuck’ in an attractor that does not maximize information entropy, but if some factor (internal or external) knocks the system out of that attractor, it will tend to end up in a different attractor with more micro-states and higher informational entropy [38,49]. Such a state tends to be more persistent because the ‘entropic barrier’ to leaving it is higher, typically taking longer to cross. Overall, the system will tend to keep evolving (ever more slowly) towards a most stable state (with the highest informational entropy). This entropic mechanism provides a direction of movement through the phase space (or configuration space) of a system. It has been described as ‘sequential selection with memory’ [38], although I think just ‘sequential selection’ is best applied to this case. Here the ‘selection’ is between (potential) macro-states of the system and the tendency towards increasing informational entropy translates into a tendency to ‘select’ progressively more diverse and stable macro-states. This entropic mechanism does not require biology and is more general than natural selection.

The resulting ‘evolution’ has been demonstrated in a model of the gradual penetration of the external magnetic field into a type II superconductor [49], and in models of evolutionary ecology including ‘Tangled Nature’ [49]. It can explain why food web models with random additions of species tend towards increasing size and stability [51,52]. This result particularly struck W. D. (Bill) Hamilton [20], who with Peter Henderson developed a ‘Dam world’ model (unpublished) that allowed (randomly added) members of an aquatic food web to build or destroy a dam that determined their ecosystem size (carrying capacity). On average ‘Dam world’ tended towards a larger and more stable system. The ‘greenhouse world’ model of networks of species and resources displays similar behaviour [53]. So too does the ‘Flask model’ of evolving microbial ecology, which tends towards increasing population and nutrient recycling and reduced extinction rate over time [26]. In its spatial variant, it also tends towards environmental regulation over time [28,29].

The mechanism has been most clearly elucidated for the Tangled Nature model (TNM), an agent-based model of co-evolutionary interaction between different species, with stochastic birth and death processes and the option for new species to be generated by mutation. In recent extensions to the TNM, agents can affect the environment and its overall habitability by altering the carrying capacity (i.e. the size of the ‘box’) up or down [37,38]. The model can be initialized with different numbers of individuals and species. Internal fluctuations can cause total extinction, but this is less likely to occur if the population is larger. The TNM typically finds an attractor that exhibits a ‘core’ of a few populous species with synergistic interactions and a ‘cloud’ of numerous weakly interacting species of low population density. The core typically tends to improve the environment (carrying capacity) as cores that degrade the environment are less populous and therefore tend to be more vulnerable to disruption. Importantly, systems starting with a higher diversity of species (but the same total initial number of individuals) tend to end up with higher population and habitability, because better mutual reinforcement between species and better overall regulation can be achieved when there are more species to ‘choose’ from. (There are a greater number of ways to produce a more productive ‘core’.)

Without a source of novelty in the TNM, there is no scope for biosphere-level evolution (towards a larger ‘box’). Allowing mutation (a source of new micro-states) enables the possibility for a new species to arise that can disrupt a stable core. Disruption can be parasitic causing the core to collapse, or it can be symbiotic causing the core to rearrange. In either case a ‘quake’ occurs, and the system undergoes abrupt change until it finds a new attractor. Importantly, more populous, diverse and habitable cores are harder to disrupt because it requires a narrower set of features of an invading species to achieve the required growth to cause disruption (there is a higher ‘entropic barrier’). There tends to be an overall trend in the macro-state towards higher population, diversity and habitability, with longer waiting times between quakes, as successive quakes begin from a higher diversity base. If environmental conditions are slowly externally forced to become less favourable this tends to be counteracted [39]. Adding external perturbations that create hostile conditions leads to more and different ‘quakes’—some end in disaster, but in general perturbations enable the finding of successively more populous, diverse and stable states (enhancing the ‘entropic ratchet’) [54].

The general entropic ratchet mechanism applied to biology has been called ‘Entropic Gaia’: co-evolutionary systems evolve in the direction of increasing information entropy, corresponding to greater biomass, diversity and life-enhancing abiotic interactions [37,38]. All of this comes without invoking (natural) selection or adaptation. ‘Tangled Nature’ and the other models mentioned above that demonstrate this behaviour [26,28,29,51–53] suggest that for a single system to exhibit improved stability (and size/diversity) of its macro-state over time it is sufficient to be comprised (in its micro-states) of a (diverse) population of things that retain their properties over time, exhibit variation and have a (random) source of variation. The ‘things’ do not have to be biological [49], but if they are this invites an alternative path to explaining what is going on—in terms of selection among the ‘things’ at the lower level. In other words, to turn to the favoured explanatory framework of biology—some form of natural selection.

## 4. The biological path to explaining Gaia

Natural selection is a variational (rather than transformational) evolutionary process. By definition, a single Gaia cannot evolve by natural selection acting at the system level. If Gaia is part of an imagined population of non-interacting biospheres then ‘selection’ based on differential survival alone can filter out the less ‘fit’ variants and leave only those with persistence enhancing properties [31]. Biospheres that by chance have some persistence-enhancing properties, or acquire them through a random walk (whereby some instances will lead to improvement), come to dominate the reduced population at later points in time—simply because those that do not go extinct [36]. This is an example of stability-based sorting [55], but it does not explain any *tendency to acquire* persistence-enhancing properties [37,38]. From this differential survival mechanism alone, the fact what we find ourselves in a persistent Gaia can be attributed to the weak anthropic principle (we would not be here to observe it were it not the case). To explain a tendency for Gaia to acquire persistence-enhancing properties over time we need a mechanism *within* the system.

The tendency towards increasing informational entropy is such a mechanism, but its ingredients, seen in the models noted above, can be recast in the form of a very general recipe for variational selection among parts of a complex system:

— there is a population of entities exhibiting variation in their properties (variation);
— those variations in properties lead to differential changes in frequency of the variants over time and/or environments (differential ‘fitness’);
— there is some continuity of those differing properties over time (transmission continuity).

This formulation, inspired by George R. Price [56], is written as a generalization of Lewontin’s canonical recipe for natural selection [57] (see below). It dispenses with reproduction but retains survival/persistence—following others [31,34,58–61]—and it generalizes heritability to temporal continuity of properties [62]. Both Price and Lewontin would (I think) deny the recipe qualifies as ‘natural selection’, but Price recognized it as selection [56]. I am not concerned about the labelling—I am just concerned with what it can explain. In this formulation it could apply to abiotic, biotic or mixed entities (like nutrient cycles or ecosystems).

Conventional natural selection following Lewontin’s recipe [57] can be seen as a special case of this more general selection recipe that applies at some lower level(s) within Gaia:

— different individuals in a population have different morphologies, physiologies and behaviours (phenotypic variation);
— different phenotypes have different rates of survival and reproduction in different environments (differential fitness);
— there is a correlation between parents and offspring in the contribution of each to future generations (fitness is heritable).

Any evolution of Gaia’s properties can be understood as due to a mix of selection at lower levels and any changes in properties of entities at lower levels. This can be formalized by recursively applying the Price equation across multiple levels [34]. The Price equation partitions a mean change in character/property in a population of entities between two times into the sum of contributions of selection and ‘transmission-bias’—meaning changes in properties over time. As Gaia is a single entity there is no selection term at the Gaia level, only transmission-bias [34]. However, in multi-level selection, what is deemed transmission-bias at one level is rewritten as due to the combination of selection and transmission-bias at the level below [24]. Thus, changes in Gaia’s properties over time can be (equivalently) understood as due to selection and transmission-bias at levels below [34].

The question becomes: what are legitimate lower levels to consider? If we allow for generalized persistence-based selection, a case can be made for ecosystems [28], nutrient cycles [63] or clades [64] at the next level, undergoing differential growth/spread and death [34]. These in turn may be seen (less controversially) as comprising reproducing species or organisms. Ultimately at the base level sit genes as the fundamental replicators. In David Hull’s formulation, replicators are contained within ‘interactors’ whose differential persistence and proliferation cause the differential perpetuation of the replicators that produce them [43,65]. There is no requirement for interactors to reproduce, but they do need to have differential ‘fitness’ and sufficient continuity of their differing properties over time. What counts as ‘sufficient’ continuity is a critical question. Some authors just assert that a particular situation has ‘too many parents’ [43,66], but this should be assessed and resolved mathematically and/or empirically.

The formulation of selection ‘recipes’ is a rather top-down approach, whereas the biological explanatory path typically reasons from the bottom up. To help resolve at what levels selection can function and what it can achieve, one can build models capturing the key ingredients of the recipes above, and of life-environment coupling, to see what (if any) Gaia phenomena the models can reproduce (while taking care to consider other mechanisms that may be at work). The ‘Flask’ model took this path [26,28,29,67], representing populations of asexually reproducing agents (thought of as ‘microbes’) each with a genetic code (with the potential for mutation) that determines their metabolism (i.e. their uptake and excretion of material resources, termed ‘nutrients’), their effect on other environmental variables (e.g. temperature, pH, salinity) and their preferred environmental conditions. Organisms cannot excrete what they consume or consume what they excrete, and effects on the environment are by-products of metabolism (rather than costly altruistic acts) that are proportional to growth rate. There is a prescribed (chemical) source of free energy to the system, and it is lost as waste heat associated with metabolism, thus setting a (theoretical) carrying capacity of the system. This is unlike Gaia, or other models [37,38], where carrying capacity may endogenously vary (figure 2). The ‘microbes’ can be placed in a single well-mixed fluid environment with steady inputs and outputs (a ‘flask’ like a chemostat) [26,68] or in a heterogeneous global environment made up of several well-mixed local environments (flasks) with some network of mixing between them [28,29].

**Figure 3.**
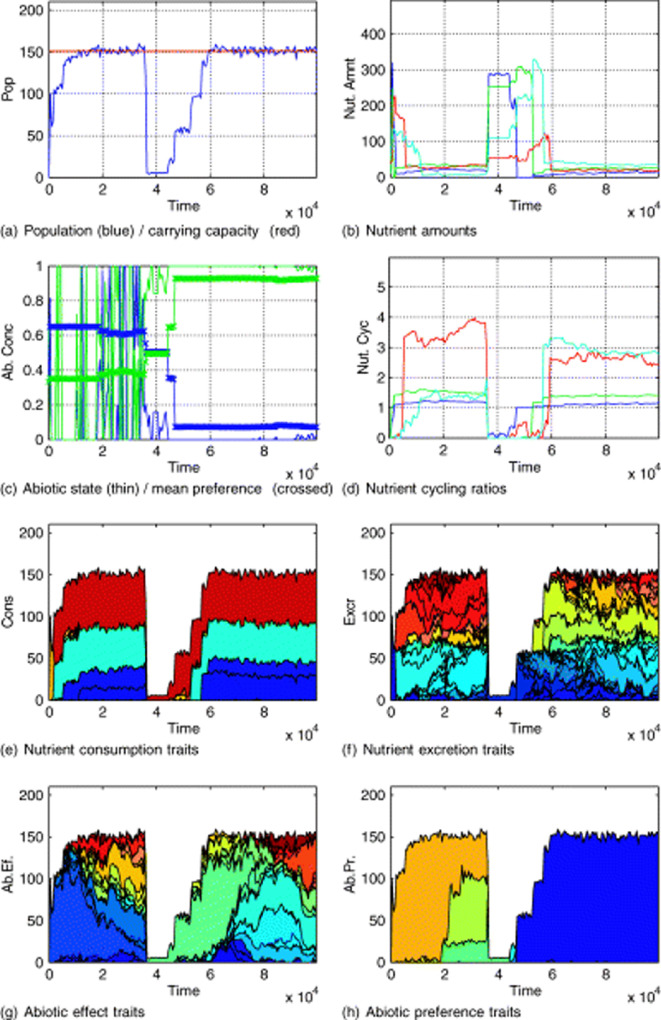
An example run of the single Flask model where strong feedback on abiotic variables causes population collapse then the system recovers—reproduced from Williams & Lenton [26]. (a) Population (variable) and carrying capacity (fixed), (b) nutrient levels, (c) state of abiotic variables (thin lines) and mean preference for them in the population (crossed lines), (d) nutrient cycling ratios, (e) nutrient consumption traits, (f) nutrient excretion traits, (g) abiotic effect traits and (h) abiotic preference traits. Efficient nutrient recycling emerges (d) supporting the population at carrying capacity (a), but environmental feedback causes a sudden population collapse. The population subsequently recovers (a), and nutrient recycling is re-established in a similar (but not identical) regime (d), with a similar balance of nutrient consumption traits (e), but there is a new stable state for the abiotic environment (c), and a new dominant allele for the abiotic preference trait (h).

In a single well-mixed environment, increasingly effective recycling of nutrients is a robust outcome. This involves natural selection of nutrient consumption traits—those organisms most effective at consuming available nutrients are the fittest and as a by-product act to close recycling loops, supporting population growth to carrying capacity [26]. Recycling can be disrupted by effects on the (non-nutrient) abiotic environment causing novel selection on environmental preferences. Sometimes a rapid (biotically driven) change in the environment causes nutrient cycling to crash and then recover (figure 3). Occasionally there is total extinction when some ‘rebel’ organisms accessing underutilized resources drive such rapid environmental change that adaptation cannot keep up. However, ensemble simulations show an overall trend of declining extinction rate over time [26], consistent with the entropic Gaia mechanism: by drawing down nutrients to very low levels and effectively recycling them, the community provides less opportunity (a higher entropic barrier) for a mutant to achieve rapid growth and disrupt the environment.

Recycling is a communal property and the nutrient cycles that arise in the model can be considered as interactors forming a population subject (in a single well-mixed flask) to persistence-based selection (following the general selection recipe above). The set of possible cycles and their topology is relatively small and determined by chemistry. In the model it is a prescribed ‘artificial chemistry’, but the real sets of possible cycles of e.g. C, N, P and S are also finite and relatively small. There are correspondingly relatively few nutrient consumption traits (metabolisms) and associated genes that make up the cycles (in the model and in reality). In the model, these metabolisms are ‘re-produced’ after population crashes—meaning a different set of organisms containing the same underlying genes assume the same cycling role (albeit in slightly different proportions) (figure 3). The cycles thus have some continuity of their properties over time, and they represent attractors that can be re-found through sequential selection if they are (temporarily) lost.

In a heterogeneous environment of multiple connected flasks, there is also the possibility of selection based on differential spread of entities. The regulation of (non-uniform) environmental variables (like temperature) tends to emerge and persist, whether the organisms have a shared, fixed environmental preference [28] or their preferences adapt [29]. This can maintain habitability when otherwise, in the absence of Life, the environment would have become uninhabitable [28]. When the environment is abruptly perturbed, the average effect of Life on the environment tends to oppose the perturbation—indicating selection [28]. This selection is not at the individual level (where environment effect traits remain diverse) as selection cannot occur within a well-mixed flask (uniform environment) when environmental preferences are shared (because environmental effect traits are selectively neutral at this level) [28]. But selection does empirically occur between ecosystems. The mechanism is that environment-improving communities which have a correspondingly higher population density tend to spread at the expense of (less dense) environment-degrading ones [28]. This occurs when the environment (rather than nutrients) is limiting to growth (and nutrient uptake traits become correspondingly diverse), hence it is not an instance of selection of nutrient cycles. Basically, positive feedback cycles between collective growth and the environment out-spread negative feedback cycles.

This is another instance of the generalized selection mechanism above, but in this case, it is whole ecosystems that function as interactors, rather than specific environmental feedback cycles. There are numerous ways of achieving environmental positive feedback and numerous diverse traits involved in instances of achieving it (as there are for negative feedback) so it is not ‘re-produced’ in the way that nutrient cycles are. Intervals of ecosystem-level selection of collective environmental effects then tend to return the system to a nutrient-limited regime where nutrient cycles are (again) the interactors (and the state of the abiotic environment can undergo a random walk until it again becomes limiting to growth).

In the Flask model, ecosystems do not have ‘too many parents’ [43] to be subject to generalized selection. Despite communities not being transmitted intact in one go from one flask to another, there is sufficient continuity of their composition across space and time for ecosystem-level selection for environment improvement to occur. The quality of environmental regulation is maximized, and the likelihood of global extinction minimized, at intermediate mixing rates, which maintain spatial heterogeneity across the global system. Extinction likelihood is also reduced by increasing the number of flasks (at a given mixing rate) which increases heterogeneity. Increasing spatial heterogeneity can be seen as increasing the number of micro-states of the system and the chance of getting an environment-improving macro-state that can spread, consistent with the entropic Gaia mechanism.

In a variant of the model where closing a resource recycling loop is assumed to incur an altruistic cost (itself a debatable assumption), cycle-level selection can override individual-level selection that favours ‘cheating’ thanks to denser recycling ecosystems spreading at the expense of less dense non-recycling ones [27]. Here again it is appropriate to think of the nutrient cycles as ‘interactors’ that tend to spread and persist. Returning to a simplified non-spatial (homogeneous) model demonstrates conditions under which persistence-based selection of closed nutrient cycles can overwhelm cheating [35]. Latest work further shows that strong covariation between ‘internal’ nutrient cycle properties and their ‘external’ environmental effects provides a basis for distinguishing cycle-level persistent-based selection from genotype-level selection [69]. This is analogous to the covariation between fitness and traits in conventional natural selection [69].

## 5. Predictions and tests

As the model results discussed in the preceding sections illustrate, the physical and biological explanatory paths to Gaia are converging. One can interpret the same phenomena in either way. Gaia’s acquisition of self-stabilizing and resource recycling properties can be explained by the tendency of complex dynamical systems to find an attractor, and for increasing informational entropy to produce a positive trend towards more productive, diverse and stable attractors. Equally, it can be explained in terms of sub-global persistence- and/or spread-based selection of ecosystems and resource cycles, with conventional natural selection also playing a role in closing nutrient cycles. The sheer intensity of resource recycling observed for some elements [1] may go beyond what entropic Gaia can explain and be a candidate ‘adaptation’ from multi-level selection. The models, although deliberately general, are not proof of the efficacy of the mechanisms, but they make testable predictions. Here I focus on testing entropic Gaia as the most general mechanism.

The entropic Gaia model predicts that on average the productivity, diversity and stability of a biosphere will increase asymptotically over time, as the time between endogenously generated ‘quakes’ (reorganizations) of the biosphere increases (and the likelihood of them causing total extinction declines). In any given instance of a biosphere, one should expect steps ‘backwards’ as well as ‘forwards’ in an overall trend (and endogenously generated extinction may occur, albeit with declining likelihood over time). We can apply these predictions to two cases: the specific case of Earth’s history and the general case of the search for life on exoplanets. We need a null model to test against, which I take to be one of no innate tendency of biospheres towards more productive, diverse or stable outcomes. We should also consider the effect of any change in frequency of exogenous perturbation.

Starting with the *n* = 1 case of Earth’s history, we must be careful to account for the bias introduced by our existence (i.e. the weak anthropic principle). However unlikely it is for chance to produce a highly productive and persistent biosphere capable of supporting self-aware observers, we must inhabit such a biosphere to be able to observe it (‘observer self-selection’ [30]). A suitable Bayesian null model already exists: the ‘critical steps’ model [70,71] predicts there were roughly four innately rare, contingent steps on the path to observers (us) that were roughly *evenly spaced*, and are argued to have involved reorganizations of the biosphere [12]. More generally, as the null model presumes no innate tendency towards greater stability, the distribution of all sizes of endogenous ‘events’ is expected to be invariant over time. Any changes in the observed size-frequency distribution of endogenous events thus offers the potential to distinguish between the entropic Gaia and null models—with the caveat that any trend in exogenous perturbations needs to be considered.

Turning to the actual record, recent molecular phylogenetic results endow the last universal common ancestor (LUCA) with a complex chemoautotrophic metabolism and push its origin back towards the origin of habitable conditions on Earth approximately 4.2 Ga [72]. A lot of metabolic innovation had apparently happened rapidly by then, since the origin of life (figure 2). Known metabolisms continued to accrue relatively rapidly thereafter, particularly 3.5−3.0 Ga, but then at a declining rate [73]—consistent with entropic Gaia. The inferred chemoautotrophic hydrogen-fuelled biosphere at the time of LUCA, even if it had acquired efficient hydrogen recycling, is estimated to have been orders of magnitude less productive than the present one [74,75]. Major increases in biosphere productivity occurred in the billion years thereafter, with the origin of different forms of anoxygenic photosynthesis and of the recycling of their associated electron donors [74,75]. Then the origin of oxygenic photosynthesis approximately 3 Ga [76] and of associated recycling of limiting nutrients likely brought biosphere productivity towards the present order of magnitude by approximately 2.7 Ga [77]. There was a marked delay to the ‘quake’ of the Great Oxidation approximately 2.4 Ga due to slowly changing Earth system forcings and feedbacks determining this tipping point [78]. There is little sign of it triggering a burst of metabolic innovation, as most metabolisms were already in place [73]. Since then, the same key metabolism (oxygenic photosynthesis) has been incorporated in more complex life forms (algae and plants). Debate about the productivity, size and diversity of the Proterozoic biosphere [3,79,80] largely boils down to how (in)efficient resource recycling was in that interval [81,82]. Finally in the Palaeozoic, the rise of land plants and associated resource recycling roughly doubled global productivity to the modern level, increasing oxygen levels and cooling the climate [83,84]. The overall picture is that metabolic evolution and gains in productivity progressively slowed down over time, consistent with the entropic Gaia model.

It is hard to say whether stability has increased over time, partly because there is no sedimentary rock record until approximately 3.8 Ga, partly because exogenous perturbation has changed over time, and partly because of the difficulty of distinguishing whether a given event has an endogenous or exogenous cause (or both). ‘Snowball Earth’ events bracketed both the beginning and end of the Proterozoic, suggesting there was still considerable instability approximately 720–635 Ma, but it is unclear if these were endogenously or exogenously triggered. Since then, the carbon cycle appears to have stabilized in the basic sense that the amplitude of variations in the carbonate carbon isotope record shows an overall decline [85]. The advent of readily fossilized macroscopic life also makes it easier to resolve trends in diversity and extinction rates—albeit with the caveats that the Phanerozoic only represents approximately 13% of the history of Life and macro-biota only a fraction of Life. Nevertheless, background extinction rates are well known to have declined and diversity to have increased [86]. Of the big five Phanerozoic mass extinctions, the first two have been hypothesized to be associated with changes in biogeochemical cycles triggered by the rise of land plants [83,87], while the latter three are all attributed to either volcanic [88,89] or meteoritic [90] external perturbations. Overall, Phanerozoic trends appear consistent with entropic Gaia.

**Figure 4.**
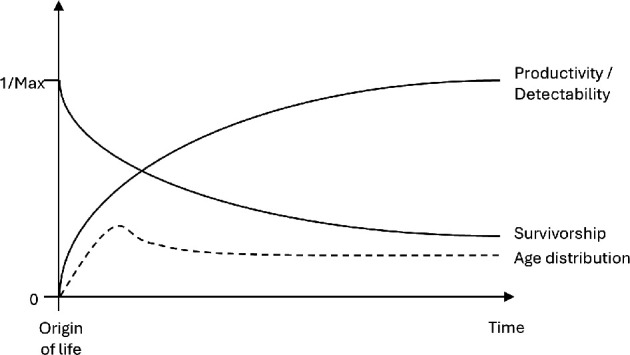
Predictions of entropic Gaia for exo-biospheres: once established, biospheres tend asymptotically towards increasing productivity, and therefore detectability, while the likelihood of endogenously generated total extinction declines asymptotically towards zero producing a stabilizing survivorship curve. The combined effect of these functions tends to produce a roughly even age distribution of detectable exo-biospheres, although their actual age is hard to constrain.

If astrobiology can succeed in increasing the sample size of biospheres to greater than one, then it can start to provide a further, statistical testing ground for entropic Gaia. The key path to detecting exo-biospheres is through their impact on the atmospheric composition of their host planet and its thermodynamic disequilibrium [91]. A more productive biosphere is more capable of transforming its host planet’s atmosphere and of creating and maintaining atmospheric disequilibrium. Entropic Gaia predicts that once established, biospheres tend asymptotically towards increasing productivity, and therefore detectability (figure 4). It also predicts that once a planet is inhabited, the likelihood of total extinction declines asymptotically over time—which translates into a declining but stabilizing survivorship curve. The combined effect should tend to produce a roughly even age distribution of detectable exo-Gaias (figure 4). By contrast, in the null case, most biospheres will die young and few are expected to achieve detectability. Entropic Gaia clearly predicts that detectable exo-biospheres are (much) more common than the null case, but the problem is quantifying *a priori* the null expectation to test against, which is influenced by uncertain factors such as the ease or difficulty of the origin of life. However, once exo-biospheres start to be detected, their spatial distribution offers a test.

**Figure 5.**
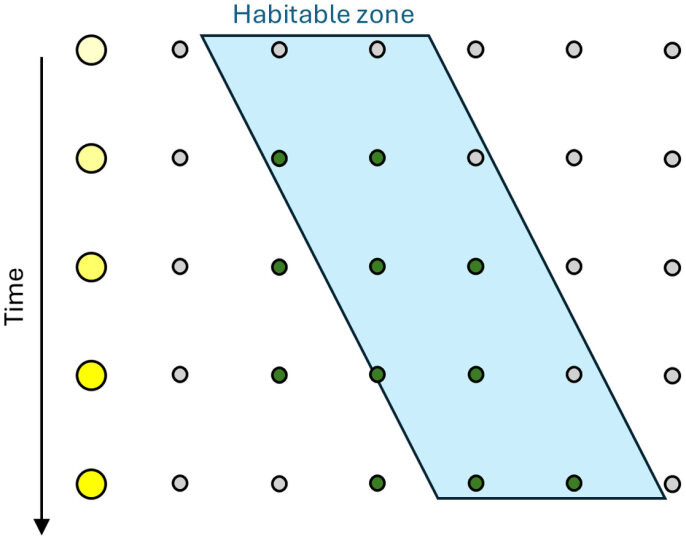
Sketch of how entropic Gaia predicts that detectable biospheres will tend to be skewed towards the inner edge and inside the abiotic habitable zone. The star (left, yellow circles) burns brighter with time moving the abiotically determined habitable zone (blue) outwards. It is orbited by several planets that are all initially uninhabited (grey circles), but Life starts relatively easily on those within the habitable zone, and the corresponding biospheres become detectable (green circles) on a timescale shorter than stellar evolution. Detectable biospheres enhance their own persistence and can maintain habitable conditions when otherwise a planet would have become uninhabitable (though not indefinitely).

It is already possible to position exoplanets spatially relative to the abiotically determined habitable zone around their parent star (where water is expected to be liquid, influenced by silicate weathering feedback). Crucially, the habitable zone around a star tends to move outwards over time, as a star on the main sequence burns steadily brighter. This means the distribution of detected biospheres with respect to position in the habitable zone is informative (figure 5). The null expectation is that detected biospheres will be evenly distributed across planets regardless of position in the habitable zone, because these biospheres are typically short-lived (compared with the timescale of stellar evolution). By contrast, if entropic Gaia is at work, then by prolonging biosphere lifespan to time scales of stellar evolution [39], the distribution of detected biospheres will skew towards the inner edge of the habitable zone, and biospheres may be found inside the (abiotically defined) habitable zone, because persistent Life has the potential to maintain habitable conditions when otherwise an abiotic planet would have become uninhabitable [39,92,93]. This reasoning is robust to not knowing the age of a planet, or when life originated, because even if the sample contains a wide range of exo-biosphere ages, including young biospheres that are indistinguishable from the null, if entropic Gaia is correct there should still be some older biospheres that skew the spatial distribution. If life were in general very slow to evolve then this would also skew the distribution of detected biospheres towards the inner edge of the habitable zone—although not within it. However, the case of the Earth suggests that life starts easily (as it appeared very soon after conditions became habitable).

There are many caveats in this simplistic treatment. For example, a steep early decline in meteoritic bombardment is an expected feature of the process of planet formation. In entropic Gaia, while exogenous perturbations may aid the ‘search’ for more stable attractors, concentrating them early when the system is less stable poses a greater risk of causing total extinction—altering the survival curve. More fundamentally, if the abiotic habitable zone theory is flawed, then the critical inference of a biosphere being within the habitable zone may become unreliable. Clearly, a more careful Bayesian approach to the problem is warranted—the intention here is simply to indicate the potential for exo-biosphere discoveries to also test entropic Gaia [39].

## 6. Conclusion

The Gaia phenomenon, whereby Life on Earth has tended to increase its own flourishing and persistence—through intense global cycling of essential elements, and the stabilization of atmospheric composition, climate and aspects of ocean composition—can be understood in two complementary ways. Following a physical explanatory path to Gaia highlights a very general mechanism whereby in a complex system containing a source of variation and some ‘memory’ of past states, the most probable trajectory is towards a state of greater size, diversity and stability. This tends to be reached through a series of punctuated equilibria where the waiting time between system reorganizations (‘quakes’) increases over time, as the system becomes progressively harder to disrupt. This ‘entropic Gaia’ mechanism can explain self-stabilizing properties of the Earth’s biosphere, and some of its internal cycling, without invoking (natural) selection or adaptation. By contrast, most of the recent literature has followed a biological explanatory path, productively seeking to generalize natural selection to explain key Gaia phenomena in terms of persistence- and/or spread-based selection among its constituent parts. Models show that the resulting generalized selection recipes can be sufficient to explain key Gaia properties (whether or not these qualify as ‘adaptations’). Notably, spread-based selection among ecosystems can produce regulation of heterogenous environmental variables, and persistence-based selection of nutrient cycles can produce increasing cycling of some elements.

The physical and biological paths to explaining Gaia are therefore converging. Both entropy and selection may play a role in explaining key biosphere properties. However, it remains unclear where generalized selection adds some explanatory power to entropic Gaia, or *vice versa*. A useful way forward would be to synthesize the two perspectives, for example, by using models to try to disentangle their mechanistic effects. Notably, to resolve whether generalized selection can produce Gaia-scale ‘adaptations’. One candidate which may qualify as an ‘adaptation’ is the intense cycling of some elements. To explore that, one approach would be to develop a variant of the ‘Flask’ model based on known (rather than artificial) chemistry, to study the evolution of recognizable nutrient cycles. Including known thermodynamics would remove any need to make assumptions about whether there are altruistic costs or not to closing cycles. The model could be further elaborated to allow the origin of new metabolisms of free energy capture. This would enable a deeper testing of whether there is a general tendency for biospheres to endogenously increase their carrying capacity, through a mix of increasing their capture of free energy and their internal cycling of resources—as has occurred for Gaia.

Already, the ‘entropic Gaia’ mechanism alone makes distinguishable predictions from a null model of no innate tendency of biospheres towards more productive, diverse or stable outcomes. Testing these predictions on the case of Earth history, while accounting for the weak anthropic principle in the null model, provides some empirical support for entropic Gaia. If exo-biospheres start to be detected, a further testable prediction of entropic Gaia is that their spatial distribution will skew towards the inner edge and inside the abiotically defined habitable zone of their parent stars. Scientific advances are thus finally starting to test a reframed Gaia hypothesis, and thus far it has survived falsification.

## Data Availability

This article has no additional data.
